# Supplementary material for: Catabolism of Alkylphenols in Rhodococcus via a Meta-Cleavage Pathway Associated With Genomic Islands
Source: Front Microbiol. 2019 Aug 20;10:1862. doi: 10.3389/fmicb.2019.01862 (PMC6710988; doi:10.3389/fmicb.2019.01862)
Supplement: Supplementary file 1 [file Data_Sheet_2.PDF]

Catabolism of alkylphenols in *Rhodococcus* via a *meta*-cleavage pathway associated with genomic islands

Supplementary Materials

1.1 Supplementary Tables

Supplementary Table 1. Primer sequences for RT-qPCR gene targets

Gene	Strain	Primers	Sequence 3'-5'
RT-qPCR primers			
aphA	EP4	aphA_E_F4	GCG GGT GCT CTA TCT CAA TC
		aphA_E_R4	GAG TCC ATC GGA ACA GTG AAG
pheA	EP4	pheA_E_F2	CTC AGC GAT CAC CAA CTA CAA
		pheA_E_R2	GAG GAC CTT GTC GAA GAT GAA G
aphC	EP4	aphC_E_F4	GGT CAC GAA GTT GCC TGT AT
		aphC_E_R4	GTC AGG ATC GAG ATG CAG ATA G
catA	EP4	catA_E_F3	CTT TCG CCG ACA TCA TCC A
		catA_E_R3	GGA GCA ACC TTG TCC TTC TC
sigF	EP4	sigF_E_250_F	GGT TCG GAT TTC GTC TCC TT
		sigF_E_366_R	GAC AGG TGC AGT TCC TTC AT
aphA	RHA1	aphA_R_F3	GAA GGA ATA CGG CCT GAT CTT
		aphA_R_R3	GAA CAT GAA GCG GGA GAG AA
pheA1	RHA1	pheA1_R_F2	GTA CAA GGA ATC GCA GGA GAA G
		pheA1_R_R2	GTA GTG GGC GAT GAA GTT GTA G
pheA2	RHA1	pheA2_R_R2	GTA GTG GGC GAT GAA GTT GTA G
		pheA2_R_R2	GGT ACA AGG AAT CGC AGG AAA
pheA3	RHA1	pheA3_R_F1	GTA CTG GAA CCA TGC GAT CA
		pheA3_R_R1	CAG GGC GAA TTC CTT CTT CT
aphC	RHA1	aphC_R_F4	AGA CCT TCG AGT ACC AGT CA
		aphC_R_R4	CGT AGT CGC GGA ACA TCT C
dmpB	RHA1	dmpB_R_F3	GGC CAA GAACCA GTA CAT CAA
		dmpB_R_R3	ATT GTC GCC CTT GCT CAT C
catA1	RHA1	catA1_R_F5	CGA CAA CGA CGG CTA CTAC
		catA1_R_R5	TCT TCG GGT CGA GGA TCA
catA2	RHA1	catA2_R_F1	CTG TTC ACGAAG GCG AAA TG
		catA2_R_R1	GTC GAG GAC GAA GCT GTA G
sigF	RHA1	sigF_R_F1	CGA GGT TCG CCG GTA TTT
		sigF_R_R1	AGG TTT GGT AGG CGT TCT TC
Gene expression primers			
aphA	EP4	aphA_full_for	gtg tgt gtC ATA TGA TGA CGA CGC CAA CTA TTC T
		aphA_full_rev	aca cac acg gat ccT CAA CCA CGC ACG ATC TT
aphB	EP4	aphB_full_for	gtg tgt gtC ATA TGA TGA CCG ACA ACA CCC C

		aphB_full_rev	aca cac acg gat ccT CAG AAC CCC CAC GGG
aphC	RHA1	aphC_full_for	ATA TAT CAT ATG ATG AGT GAC GCT CGT TTC GAC ATC
		aphC_full_rev	tat ata GGA TCC TCA GAG CGA CGC
Deletion mutant primers			
Cloning primers			
aphA	RHA1	aphA up-F[a]	CTA GTC TAG ATC CGC GAT ATC GAC GAA CAA
aphA	RHA1	aphA up-R[b]	CGG GGT ACC AGT TAC GGA AAG CCG GAT GG
aphA	RHA1	aphA dn-F[b]	CGG GGT ACC ACA GTG CAT GTC CGA GTA CG
aphA	RHA1	aphA dn-R[c]	CCC AAG CTT CTA TTT CAC GGC GAT CGT GC
aphC	RHA1	aphC up-F[a]	CTA GTC TAG ACT CGC TGA ATT CGC TGA GGT
aphC	RHA1	aphC up-R[b]	CGG GGT ACC TTC GTG AAG AAG TCC AGC GT
aphC	RHA1	aphC dn-F[b]	CGG GGT ACC CTG CCG TGG GAG TCG TAT TT
aphC	RHA1	aphC dn-R[c]	CCC AAG CTT ATT CGC TCT TCC CCT CAA CC
Screening primers			
aphA	RHA1	aphA int-F	GTC GAG TAC GAC ACC GAC AGC G
aphA	RHA1	aphA int-R	TCC TCA CAC TCG GCG GTG GTC
aphA	RHA1	aphA scr-F	TCT GCA GCG CTG GTC GCC AAC
aphA	RHA1	aphA scr-R	CGC GAG AAC CAC CGT TAC AGA TG
aphC	RHA1	aphC int-F	GGA CAG CGA GCG CCA TCA C
aphC	RHA1	aphC int-R	GAG GGT GCG TCA GAG CGA CG
aphC	RHA1	aphC scr-F	GCAT GTT CAT CGG CGG TTC G
aphC	RHA1	aphC scr-R	GGA AGG CGA CAC CGT CAT CCA G

[a]underlined bases show XbaI restriction site

[b]underlined bases show KpnI restriction site

[c]underlined bases show HindIII restriction site

Supplementary Table 2. Characteristics of genomes analyzed in this study.

Genome	Assembly Acc.	Size (Mb)	GC (%)	Chromosome	Plasmids	Scaffolds	Genes	Proteins	Isolation
***Rhodococcus* rhodochrous EP4**	**GCA_003004765.2**	**5.72**	**67.9**	**linear**	**0**	**1**	**5198**	**4942**	**Agricultural compost**
Rhodococcus rhodochrous DSM43241	GCA_001646825.1	5.18	68.2	circular	0	--	4790	4585	Soil
Rhodococcus pyridinivorans SB3094	GCA_000511305.1	5.31	67.8	circular	2	--	5165	4893	Diesel waste site
Rhodococcus sp. BUPNP1	GCA_002215235.1	5.56	68.1	linear	--	89	5092	4924	Landfill soil
Rhodococcus sp. 2G	GCA_001886355.1	5.23	67.5	circular	1	--	5256	4930	Activated sludge
Rhodococcus sp. p52	GCA_000763325.2	5.41	67.9	circular	3	--	5076	4753	Oil-polluted soil
Rhodococcus biphenylivorans TG9	GCA_003288095.1	5.03	68	circular	0	--	4700	4373	PCB-contam. sediment
Rhodococcus erythropolis CCM2595	GCA_000454045.1	6.37	62.5	circular	1	--	5895	5776	Soil
Rhodococcus opacus B4	GCA_000010805.1	8.83	67.6	linear	5	--	8227	7837	Chemical plant soil
Rhodococcus jostii RHA1	GCA_000014565.1	9.7	67	linear	3	--	9256	8690	Lindane-contam. soil

Genome	Total GIs[a]	Non-overlap GIs[b]	%GI[c]	GI genes
***Rhodococcus* rhodochrous EP4**	**61**	**38**	**7.4**	**1049**
Rhodococcus rhodochrous DSM43241	35	27	8.1	482
Rhodococcus pyridinivorans SB3094	37	24	7.9	605
Rhodococcus sp. BUPNP1	9	8	7.0	331
Rhodococcus sp. 2G	50	30	8.4	863
Rhodococcus sp. p52	25	15	5.3	611
Rhodococcus biphenylivorans TG9	35	25	6.9	662
Rhodococcus erythropolis CCM2595	3	2	1.1	62
Rhodococcus opacus B4	91	72	8.5	980
Rhodococcus jostii RHA1	103	84	9.2	1104

[a]All GIs on largest replicon predicted by IslandPick, SIGI-HMM and IslandPath-DIMOB using IslandViewer4

[b]Number of GI regions with overlapping elements combined

[c]Percent of total replicon length identified as belonging to a GI

Supplementary Table 3. Phenol hydroxylases (oxygenase) proteins in EP4 and RHA1

Gene	Strain	Gene ID[a]	Product ID	Location	Best Hit[b]	% ID[c]	Ref.
pheA	EP4	RS07250	WP_059382681.1	Chr.	Chlorophenol-4-monooxygenase Q8GMG6	65	Liu et al. (2002)
pheA1	RHA1	RS11630	WP_009475043.1	Chr.	Chlorophenol-4-monooxygenase Q8GMG6	65	Liu et al. (2002)
pheA2	RHA1	RS12295	WP_011595238.1	Chr.	Chlorophenol-4-monooxygenase Q8GMG6	65	Liu et al. (2002)
pheA3	RHA1	RS35960	WP_011599019.1	pRHL1 plasmid	Chlorophenol-4-monooxygenase Q8GMG6	63	Liu et al. (2002)
aphA	RHA1	RS18785	WP_009476953.1	Chr.	Chlorophenol-4-monooxygenase Q8GMG6	63	Liu et al. (2002)

[a]Locus in EP4 (C6369_RSXXXXX); locus in RHA1 (RHA1_RSXXXXX).
[b]Characterized homologs were found using BLASTp against the PDB database.
[c]Percent identity of best hit and EP4 homolog determined by Clustal Omega alignment.

1.1 Supplemental Figures

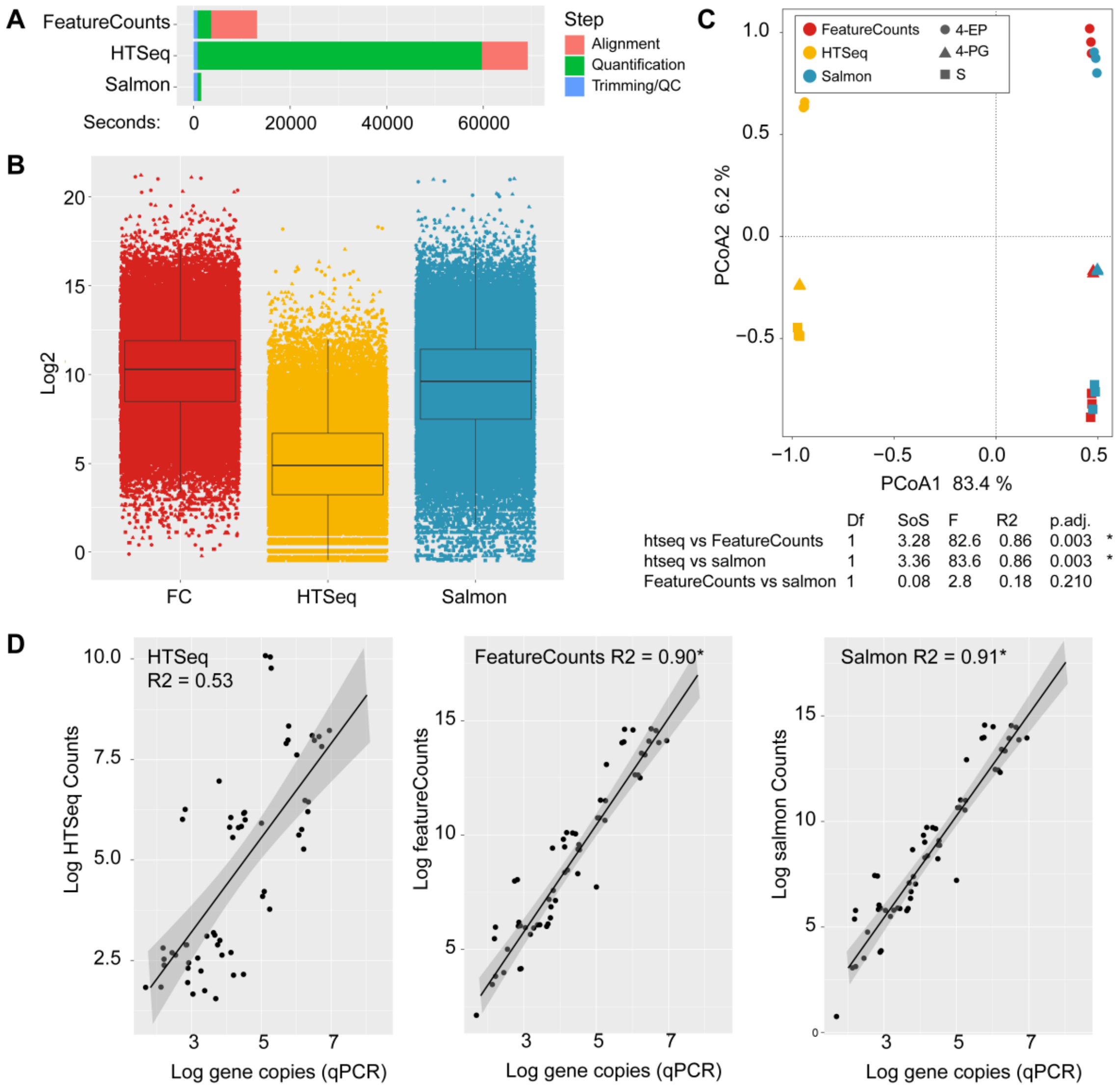

Supplementary Figure 1. Quantitative mapping of prokaryotic RNASeq reads. A) Compute time in seconds for read mapping steps for three methods of feature quantification: FeatureCounts (FC), HTSeq and Salmon. Timing benchmarks calculated on a 256 Gb, 32-core Ubuntu 14.04.5 server, running eight cores per analysis. B) Total counts produced by each method. C) PCoA ordination of read counts of all genomic features with results of pairwise PERMANOVA. D) Linear regression of RNASeq method against RT-qPCR transcript abundance showing R_{2adj}. All p-values < 0.0001). 4-EP, 4-ethylphenol; 4-PG, 4-propylguaiacol; S, succinate. Full 4-PG results can be found in a separate manuscript (Fetherolf et al., unpublished).

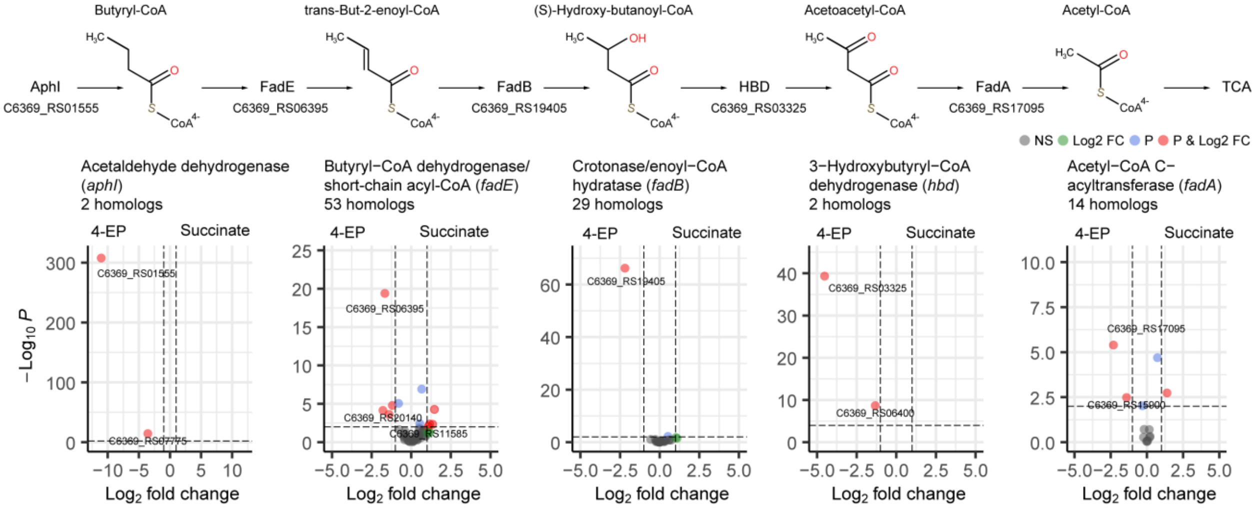

Supplementary Figure 2. Transcriptomic analysis of the metabolism of butyryl-CoA produced from 4-ethylphenol catabolism by the Aph pathway in EP4. The top panel shows the predicted pathway of metabolism showing the production of the acyl-CoA during the final step of the Aph pathway. The bottom panels show plots of -$\log_{10}$ transformed *p*-values plotted against $\log_2$ fold change for all putative homologs of the genes encoding each step of acyl-CoA metabolism during growth on 1.0 mM 4-ethylphenol versus succinate calculated using *DeSeq2*.

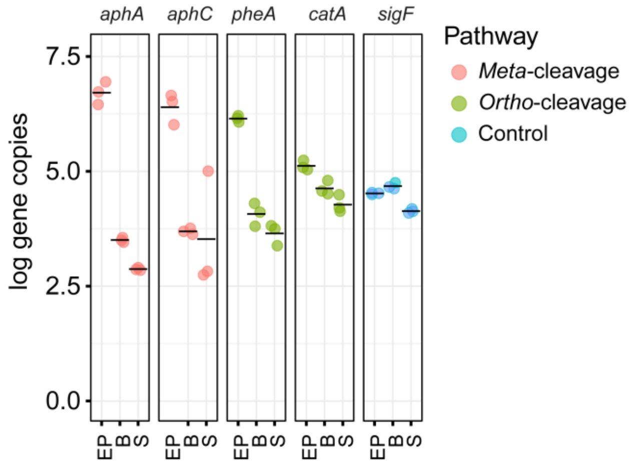

Log2 FC	*aphA*	*aphC*	*pheA*	*catA*	*sigF*
EP v S	11.5**	9.5	8.3*	2.8*	1.3**
EP v B	10.6**	9.0*	6.9*	1.6	-0.5
B v S	2.1**	0.6	1.41	1.2	1.8**

Supplementary Figure 3. RT-qPCR expression of *aphA*, *aphC*, *pheA*, *catA* and *sigF* (control) genes during growth of EP4 on 1 mM 4-ethylphenol (EP), 2 mM succinate (S) and 1 mM benzoate. N = 3, $\log_{10}$ gene copies ng^{-1} total RNA). Table shows $\log_2$ fold change and *p*-value following Bonferroni-corrected two-tailed Student's t-tests: *, $p_{bon} < 0.05$, **, $p_{bon} < 0.01$.

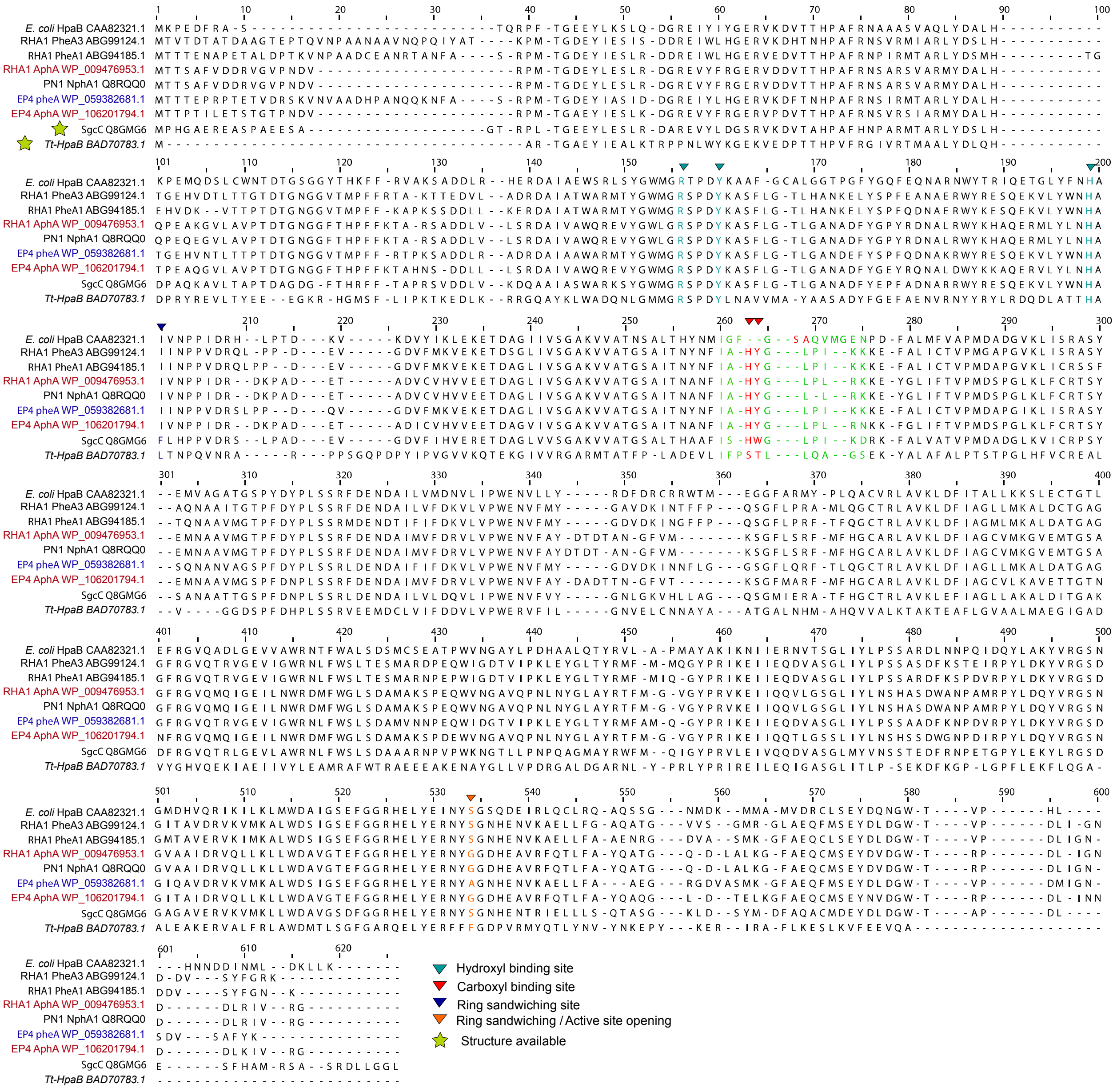

Supplementary Figure 4. TCoffee-Expresso-based structure alignment of EP4 AphA and select homolog amino acid sequences. See Figure 3 for sequence identification.

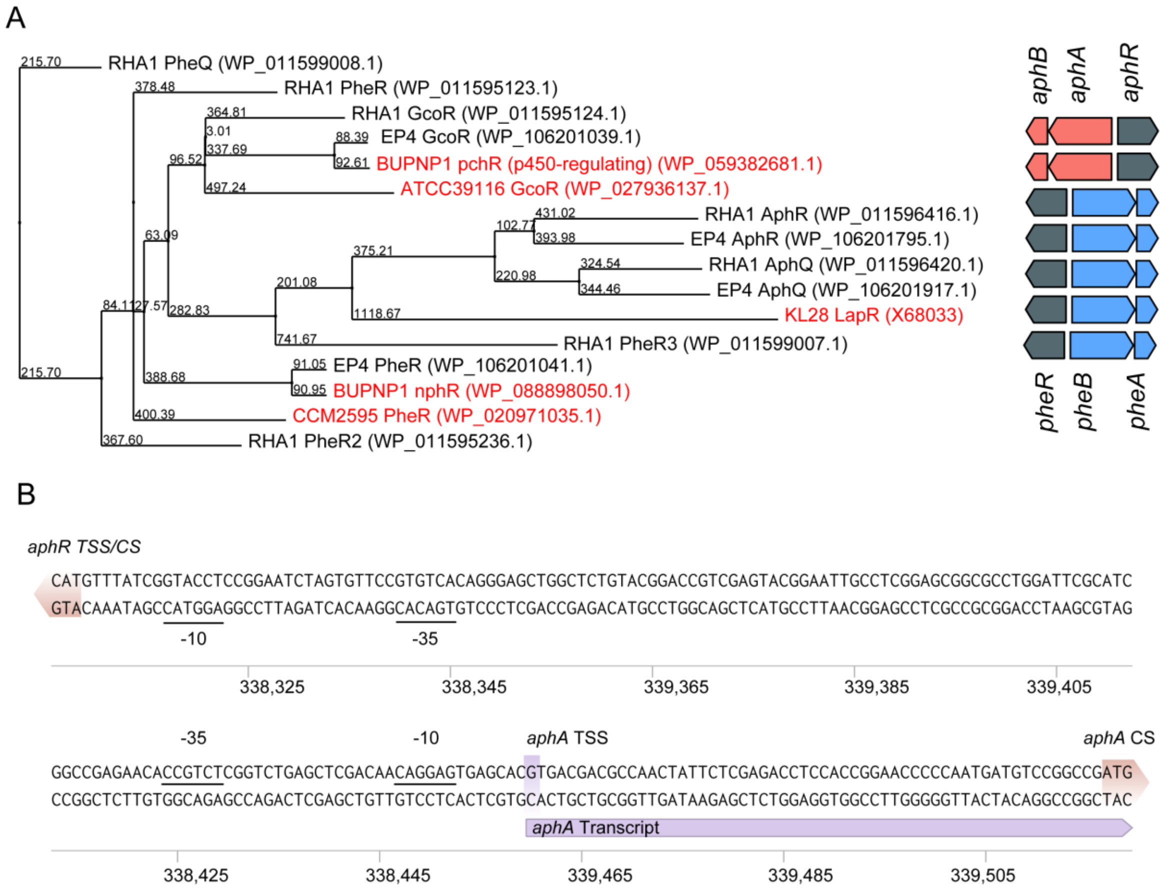

Supplementary Figure 5. A) RAxML-tree from TCoffee-aligned AraC-family transcriptional regulatory proteins. Red indicates previously identified proteins. B) Map of the *aphR*-*aphA* intergenic region showing *aphA* promotors (solid underline), *aphA* transcriptional start site (TSS) (purple) and predicted coding start (CS) (pink). Strains: ATCC39116, *Amycolatopsis* sp. ATCC39116; BUPNP1, *Rhodococcus* sp. BUPNP1; CCM2595, Rhodococcus *erythropolis* CCM2595; KL28, *Pseudomonas* sp. KL28.

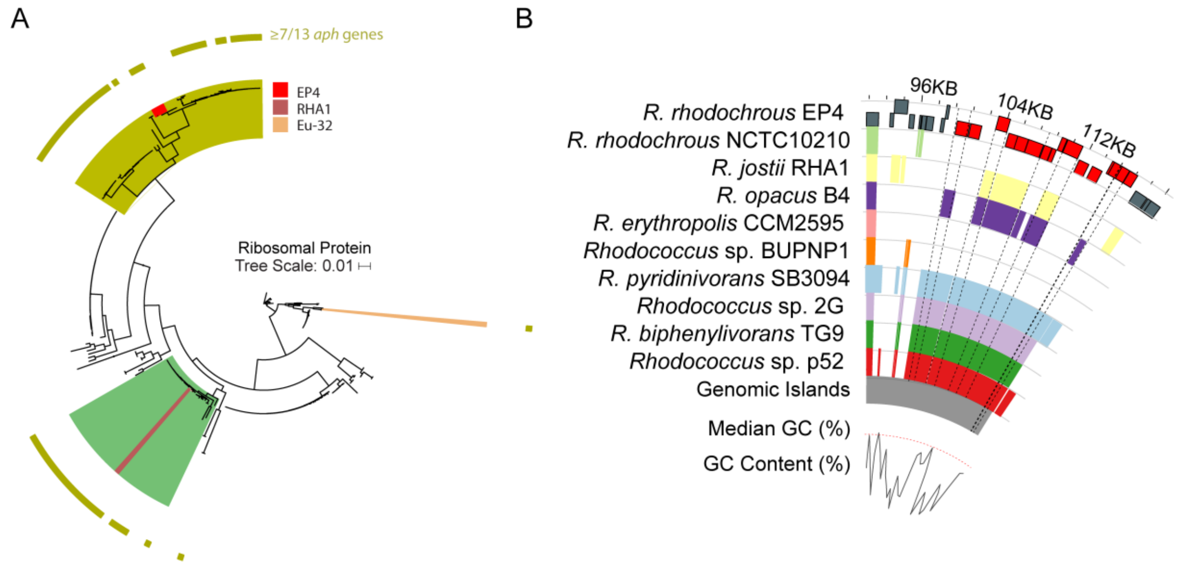

Supplementary Figure 6. The *aph* cluster in *Rhodococcus* genomes. A) RAxML phylogenetic tree calculated from MUSCLE alignment of concatenated sequences for ribosomal proteins L2, L3, L4, L5, L6, L14, L16, L18, L22, L24, S3, S8, S10, S17 and S19 from 325 *Rhodococcus* ssp. genomes showing genomes for which BLASTp evalues were < 10^{-100} for at least 7 of the 13 EP4 Aph amino acid sequences (gold boxes). Also showing the *Rhodococcus opacus*/*jostii*/*wratislaviensis* clade (green), *Rhodococcus rhodochrous*/*pyradinivorans* clade (gold), and *Rhodococcus* sp. Eu-32 (peach). B) Detail from Figure 4 showing *nucmer* alignment of select *Rhodococcus* genomes to the EP4 *aph* gene cluster including a portion of the 117-kb *aph* gene-containing genomic island predicted using IslandViewer4 (grey), showing *nucmer* aligned regions (black dashed lines), median GC content (red dashed line) and GC content in 200 bp segments (solid grey line).
