# Supplementary material for: Catabolism of Alkylphenols in Rhodococcus via a Meta-Cleavage Pathway Associated With Genomic Islands
Source: Front Microbiol. 2019 Aug 20;10:1862. doi: 10.3389/fmicb.2019.01862 (PMC6710988; doi:10.3389/fmicb.2019.01862)
Supplement: Supplementary file 2 [file Presentation_1.pdf]

Catabolism of alkylphenols in *Rhodococcus* via a *meta*-cleavage pathway associated with genomic islands

Supplemental Methods

Authors: David J. Levy-Booth[a], Morgan M. Fetherolf[a], Gordon Stewart[a], Jie Liu[a], Lindsay D. Eltis[a] and William W. Mohn[a#]

Affiliations:

1. Department of Microbiology & Immunology, Life Sciences Institute, The University of British Columbia, Vancouver, British Columbia, Canada

RT-qPCR

Gene expression was also assessed using RT-qPCR using primer sequences shown in Supplementary Table 1. Reverse transcription to cDNA used SuperScript VILO (Life Technologies) and Turbo DNase (Thermo Fisher Scientific). Cycling conditions for all genes were: 95°C for 5 min; 40 cycles of: 95°C for 30s, 52°C for 20s, 72°C for 30s (read fluorescence) on the StepOnePlus Real-Time PCR System (Thermo Fisher Scientific). Standard curves for all genes were made using 10x serial dilutions of pCR2.1-TOPO TA plasmids containing the target amplicon from 10^9 to 10^2. RT-qPCR transcript abundances were compared statistically using two-tailed Student's t-tests with Bonferroni (*bon*) correction.

Protein purification

Full-length *aphA* and *aphB* genes were amplified from *Rhodococcus* EP4 DNA and *aphC* was amplified from RHA1 DNA using Phusion polymerase. Primers (Supplementary Table 4) were designed with N and C terminal NdeI/BamHI sites respectively. Amplicons were inserted into a pET15b plasmid and inserts sequenced. The pET15b backbone contained an N-terminal histidine tag with a TEV cut site inserted between the histidine tag and the coding sequence. Constructs were transformed into *E. coli* BL21 (DE3) cells using standard protocols. Individual colonies were pick and grown overnight in 5 mL of LB containing 100 mg/L ampicillin. These cells were then used to inoculate 2 x 1 L cultures in LB and grown until OD600 was ~0.5 and induced with 0.5 mM IPTG. Cultures containing the *aphB* construct contained 200 mg/L ammonium iron citrate to facilitate cofactor incorporation. Cells were grown overnight at 25°C and harvested by centrifugation the next day. Pellets were lysed using an EmulsiFlex-C5 homogenizer (Avestin, Ottawa, ON, Canada) and centrifuged a ~26,000 x g for 20 minutes to clarify the lysate. All enzymes were purified by Ni sepherose 6 fast flow resin according to the manufacturer's protocol. The histidine tag was removed by dialyzing overnight in 20 mM Tris pH 8.0 with TEV protease. TEV protease and any uncleaved enzyme were removed by flowing the lysate over the Ni resin. The $AphA_{EP4}$ enzyme was further purified using a MonoQ 10/100 GL and an ÄKTA Purifier (GE Healthcare) using a linear gradient of 20 mM Tris pH 8.0 to 20 mM Tris 1 mM NaCl pH 8.0 over roughly 6 CV.

$AphB_{EP4}$ reductase activity

The activity of $AphB_{EP4}$ was measured by the reduction of cytochrome *c* spectrophotometrically using scanning kinetic mode on a Cary 60 spectrophotometer set to 550 nm as in (Guengerich et al., 2009). Roughly 7 nM $AphC_{RHA1}$, 40 µM bovine cytochrome *c*, and 100 µM of either FAD, FMN or riboflavin were dissolved in 300 mM phosphate buffer pH 7.7 at 25°C. The reaction was initiated with the addition of 100 µM NADH and the increase in absorbance was measured. Specific activity was calculated using ε = 0.021 mM^{-1} cm^{-1} for cytochrome *c*.

Enzyme end point assays

For end point assays, 100 µM substrate (200 µM for 4-HPA and 4-NP) in 0.5 mL 20 mM MOPS, 90 mM NaCl, pH 7.2 containing 20 µM $AphA_{EP4}$ and 2 µM $AphB_{EP4}$, were incubated overnight at 30 °C. Samples were quenched with the addition of acetic acid to a final concentration of 1%. The samples were then centrifuged at 16,000 × g for 5 minutes and filtered through a 0.2-µm syringe filter. Samples were analyzed using a Waters 2695 HPLC (Waters, Milford, MA, USA) equipped with a Luna 5 µm C18(2) column 250 × 4.6 mm (Phenomenex, Torrance, CA, USA) and a UV detector. The column was operated at

0.7 ml min^{-1} and the sample was eluted using a 16.8 ml linear gradient of 1% formic acid in H_2O to 100% methanol. Elution of compounds was monitored at 280 nm.

aphAB$_{EP4}$ transcriptional regulation

To understand how the transcriptional framework of the *aphAB* genes, we first aligned the assembled transcriptome to the EP4 genome, which provided experimental evidence of the transcription start site (TSS). Promotor prediction used BPROM (Solovyev and Salamov, 2011). PGAP gene prediction provided the coding start site (CS). Promotor sequences and AphR binding regions were predicted based on promotors in other *Actinobacteria* and alignment to sequences upstream of *Rhodococcus pheA1* (Bashyam et al., 1996; Szőköl et al., 2014). The role of AphR transcriptional regulator proteins were predicted based on position in the EP4 genome and RAxML phylogenetic analysis against characterized AraC-family proteins following TCoffee sequence alignment.

Supplemental References

Bashyam, M.D., Kaushal, D., Dasgupta, S.K., Tyagi, A.K., 1996. A study of mycobacterial transcriptional apparatus: identification of novel features in promoter elements. Journal of Bacteriology 178, 4847–4853. https://doi.org/10.1128/jb.178.16.4847-4853.1996

Guengerich, F.P., Martin, M.V., Sohl, C.D., Cheng, Q., 2009. Measurement of cytochrome P450 and NADPH-cytochrome P450 reductase. Nat Protoc 4, 1245–1251. https://doi.org/10.1038/nprot.2009.121

Solovyev, V., 2011. V. Solovyev, A Salamov (2011) Automatic Annotation of Microbial Genomes and Metagenomic Sequences. In Metagenomics and its Applications in Agriculture, Biomedicine and Environmental Studies (Ed. R.W. Li), Nova Science Publishers, p.61-78. pp. 61–78.

Szőköl, J., Rucká, L., Šimčíková, M., Halada, P., Nešvera, J., Pátek, M., 2014. Induction and carbon catabolite repression of phenol degradation genes in *Rhodococcus erythropolis* and *Rhodococcus jostii*. Appl Microbiol Biotechnol 98, 8267–8279. https://doi.org/10.1007/s00253-014-5881-6
